# Acute Exercise-Induced Set Shifting Benefits in Healthy Adults and Its Moderators: A Systematic Review and Meta-Analysis

**DOI:** 10.3389/fpsyg.2021.528352

**Published:** 2021-01-29

**Authors:** Max Oberste, Sophia Sharma, Wilhelm Bloch, Philipp Zimmer

**Affiliations:** ^1^Department of Molecular and Cellular Sport Medicine, Institute of Cardiovascular Research and Sports Medicine, German Sport University Cologne, Cologne, Germany; ^2^Division of Performance and Health (Sports Medicine), Institute for Sport and Sport Science, Technical University Dortmund, Dortmund, Germany

**Keywords:** exercise, acute exercise, physical activity, cognition, set shifting, task switching, Trail Making Test and Wisconsin Card Sorting Test

## Abstract

**Background:** Positive effects of acute exercise on cognitive performances in general inspired research that investigated the effects of acute exercise on specific cognitive subdomains. Many existing studies examined beneficial effects of acute exercise on subsequent set shifting performance in healthy adults. Set shifting, a subdomain of executive function, is the ability to switch between different cognitive sets. The results of existing studies are inconsistent. Therefore, a meta-analysis was conducted that pooled available effect sizes. Additionally, moderator analyses were carried out to identify covariates that determine the magnitude of exercise-induced set shifting benefits.

**Methods:** Medline, PsycINFO, and SPORTDiscus were searched for eligible studies. Hedges' g corrected standardized mean difference values were used for analyses. Random-effects weights were applied to pool effects. Potential moderation of the effect of acute exercise on subsequent set shifting performance by exercise intensity, type of exercise, participants' age, and type of control group were examined.

**Results:** Twenty-two studies (*N* = 1,900) were included into analysis. All aggregated effect sizes ranged from small to moderate. Overall, a small significant beneficial effect was revealed (g = −0.32, 95 % CI −0.45 to −0.18). Heterogeneity of included effect sizes was moderate and significant (T^2^ = 0.0715, I^2^ = 46.4%, (*p* < 0.0016). Moderator analyses revealed a larger average effect in older adults than for studies examining younger adults (−0.42 vs. −0.29). Light exercise (−0.51) led to larger effects than moderate (−0.24) or vigorous exercise (−0.29). Studies testing acute exercise against active control groups showed a noticeably smaller average effect (−0.13) than studies that used passive (−0.38) or cognitive engaging control groups (−0.34). Interestingly, application of resistance or aerobic exercise led to no different average effect sizes (−0.30 vs. −0.32). However, none of the tested covariates reached statistical significance.

**Conclusion:** Acute exercise improves subsequent set shifting performance. However, effect sizes are small, making the relevance for everyday life questionable. The results indicate that older adults benefit more from acute exercise than younger adults do. Light intensity exercise seems most effective while the type of exercise does not seem to influence the magnitude of effects. Research designs with active control groups show the smallest average effect, raising concerns about placebo effects.

PROSPERO registration number: CRD42019138799

## Introduction

The investigated effects of acute exercise on the cardiovascular, respiratory, musculoskeletal and other organic systems (Mathews and Fox, 1976) inspired research to examine whether acute exercise has an influence on cognitive functioning as well (Tomporowski and Ellis, 1986). After several years, studies revealed beneficial effects of acute exercise on the central nervous system. Among others, it increases prefrontal oxygenation (Endo et al., 2013), cortical activation (Yanagisawa et al., 2010), neurotrophin (Schmolesky et al., 2013) and catecholamine expression (Chmura et al., 1994), and it improves the metabolic status of cerebral neurons (Dalsgaard et al., 2004). These positive physiological adaptations to acute exercise within the central nervous system once again raised the question whether cognitive performances might also benefit as a result even more. In particular, potential effects of acute exercise on subsequent higher cognitive performances are in the focus of today's research interest. Accordingly, this meta-analysis focuses on the potential effects of acute exercise on subsequent set shifting performance. Set Shifting is a subcomponent of executive function (Pennington and Ozonoff, 1996; Miyake et al., 2000; Alvarez and Emory, 2006; Diamond, 2013) and is often used synonymously for the more common term “task switching,” which is owed to vague minor differences in the definitions of both terms (Rogers and Monsell, 1995; Rushworth et al., 2005; Ravizza and Carter, 2008; Koch et al., 2018). Set shifting implies the ability to switch between cognitive sets by altering behavior and actions due to changing conditions (Cools, 2001). In other words, being capable of moving back and forth between tasks, operations or mental sets (Miyake et al., 2000). This distinguishes set shifting from multitasking, which is defined as a cognitive process involved in performing two or more tasks at the same time (Koch et al., 2018). Set shifting was shown to predict problem solving abilities (Senn et al., 2004) and is required for social competence as well (Bierman et al., 2008).

Still, the exact neurobiological mechanisms by which the process of set shifting occurs are still unknown (Ludyga et al., 2016; Pontifex et al., 2019). However, it was shown that the prefrontal and posterior parietal cortices are mainly involved in making set shifts possible (Miyake et al., 2000; Pa et al., 2010) and that the dorsolateral prefrontal cortex is more active in exactly these situations (Meyer et al., 1998; MacDonald et al., 2000). Due to the above-mentioned neurobiological influence acute exercise has on the central nervous system in addition to the reported involvement of different cortical areas, potential effects of acute exercise on subsequent set shifting performance are assumed (Chang et al., 2012). Often examined neurobiological factors on set shifting performance are catecholamines (Arnsten, 2011) and the brain-derived neurotrophic factor (BDNF) (Nakazato et al., 2009; Gajewski et al., 2011; van der Kolk et al., 2015). Both factors are shown to react strongly to acute exercise (Knaepen et al., 2010; Schmolesky et al., 2013). It is important to note that this meta-analysis focusses on the effect of acute exercise on behavioral set shifting performance and not on potential neurobiological moderators.

Many studies exist that examine the effects of acute exercise on subsequent behavioral set shifting performance (Coles and Tomporowski, 2008; Barenberg et al., 2015; Crush and Loprinzi, 2017). The results are inconsistent. While several studies showed beneficial effects (Murray and Russoniello, 2012; Jaffery et al., 2018; Naderi et al., 2018), other studies show no effects (Frith et al., 2017) or even detrimental effects (Basso et al., 2015). In an attempt to clarify this disparity, this meta-analysis is the first to examine potential moderators that might influence the effect of an acute exercise bout on subsequent set shifting performance. Differences in the intensity of applied exercise might explain the inconsistency of results. It is assumed that intensity is a crucial covariate that determines quality and magnitude of physiological adaptations to acute exercise within the central nervous system (Knaepen et al., 2010; McMorris, 2016). Another potential moderator of the effect of acute exercise on subsequent set shifting is the type of exercise. Resistance and aerobic exercise represent distinct spectra of exercise defined by different physical demands, e.g., cardiovascular, metabolic, or musculoskeletal (Pontifex et al., 2009).

Apart from features of the exercise regimen, characteristics of examined participants might also explain inconsistency of existing studies that examined the effect of acute exercise on subsequent set shifting performance. Executive function performances are considered to decrease during older adulthood (Brydges et al., 2013; Pettigrew and Martin, 2014). On the contrary, young healthy adults seem to be at the peak of their cognitive performance (Zelazo et al., 2004). There is evidence, which indicates that individuals with lower baseline cognitive performance benefit most from acute exercise (Sibley and Beilock, 2007; Drollette et al., 2014; Dimitrova et al., 2017). Therefore, it is conceivable that participants' age plays a moderating role in exercise-induced set shifting benefits.

Finally, methodological features might explain variability of reported effects of acute exercise on subsequent set shifting. Particularly, the type of applied control group was discussed. It was hypothesized that in research designs with passive control groups expectation-driven placebo effects might play a role (Oberste et al., 2019).

In this systematic review and meta-analysis, an overview of the current research of the effects of acute exercise on subsequent set shifting performance in healthy adults is provided. Moreover, moderator analyses are carried out to identify covariates that determine the magnitude of exercise-induced set shifting benefits. The intensity of the applied exercise, the type of exercise, participants' age, and the type of control group are checked for moderation of the effect of acute exercise on subsequent set shifting performance.

## Methods

This meta-analysis followed the PRISMA reporting guidelines (Moher et al., 2009). We provide the PRISMA checklist in the Supplementary Material to this article. The key features of this review were registered in advance on PROSPERO. (registration number: CRD42019138799).

### Trial Eligibility Criteria

Studies were eligible for this review if they were published peer-reviewed in English language. The year of publication was not a limiting factor. Eligibility criteria for this review were defined based on the PICOS approach:

#### Population

Studies were included if they investigated healthy adults. Adult was defined as 18 years of age or older. Studies were excluded if they investigated participants under 18 years of age, ill individuals, or animals.

#### Intervention

We included studies that applied a single aerobic or resistance exercise session of 5–60 min duration and of a light, moderate or vigorous intensity. Resistance and aerobic exercise were understood as defined by the American College of Sports Medicine (ACSM). They define aerobic exercise as any activity that uses large muscle groups, can be maintained continuously and is rhythmic in nature (Pescatello et al., 2014). Resistance exercise includes free weights, machines with stacked weights or pneumatic resistance, and even resistance bands (Pescatello et al., 2014). The division into light, moderate and vigorous intensity was based on the definitions by Norton et al. (2010) for the aerobic exercises (Norton et al., 2010), and by Jovanovic (2013) for the resistance exercises (Jovanović and Flanagan, 2014).

#### Comparison

Trials were included if they compared the effects of acute exercise against a control condition that did not exceed the threshold for light intensity as defined by Norton et al. (2010) and Jovanović and Flanagan (2014). Studies were excluded if they compared only the effects of two exercise treatment groups without a control group.

#### Outcome

Studies were included if they measured behavioral set shifting performance as a primary or secondary endpoint. Studies were excluded if they measured behavioral set shifting performance only during exercise.

#### Study Design

Studies were included if they applied a randomized crossover study design or a randomized controlled trial study design. Studies with other designs were excluded.

### Cognitive Assessments Studies

using one of the following neuropsychological tests for assessing set shifting performance were included in this meta-analysis. The Trail Making Test (De Oliveira-Souza et al., 2000; Moll et al., 2002; Sánchez-Cubillo et al., 2009), the Wisconsin Card Sorting Task (Konishi et al., 1998; Gamboz et al., 2009), the Switch Trial test (Rogers and Monsell, 1995), the Dimension Switching Task (Boucard et al., 2012), the Plus-Minus Task (Boucard et al., 2012), the More Odd Task (Chen et al., 2014), the Task Switching Test (Dai et al., 2013) and the Visual Switch Task (Tomporowski and Ganio, 2006) are defined as valid measures of set shifting performance. A detailed description including the instructions for each of the above stated tests can be found in the Supplementary Material.

### Search Strategy

The literature search was last updated on the 25th of May 2020, using the electronic databases Medline, SPORTDiscus and PsychINFO via EBSCO host. To determine relevant studies, the terms “exercise,” “sport,” “physical activity,” “physical exertion,” “physical training,” “running,” “jogging,” “walking,” “bicycling,” “strength training” were combined with “cognition,” “executive function,” “set shifting,” “reaction time” and “attention.” The exact search algorithm can be found in the Supplementary Material to this review. The literature search was conducted independently by two members of the review team (S.S. and M.O.). Inconsistencies were solved by discussion.

### Outcome Measures and Data Extraction

For included studies, the number of participants in each group, as well as the group means and standard deviations of measures of set shifting performance were extracted. Moreover, we extracted information of potential moderators (exercise intensity, type of exercise, participants' age, and type of control group). If measures were not reported in the full-text of the study, the corresponding author was contacted, and the raw data was requested. If the results were presented in form of a graphic, data was extracted using the WebPlotDigitizer (Tsafnat et al., 2014). For studies with multiple treatment arms that did not deviate regarding here tested moderators, the data was summarized according to the methods suggested by Higgins and Deeks (2011). In case of a repeated testing of the participants, the measurement taken first was used. Data extraction was assessed independently by two members of the review team (S.S. and M.O.). Disagreements were solved by discussion.

### Risk of Bias Assessment

To assess methodological quality, two authors (S.S. and M.O.) independently rated each included study according to the Physiotherapy Evidence Database (PEDro) scale. The PEDro scale consists of the following 11 items: (1) eligibility criteria, (2) random allocation, (3) concealed allocation, (4) baseline comparability, (5) blinding of subjects, (6) blinding of therapists, (7) blinding of assessors, (8) completeness of follow-up, (9) intention-to-treat-analysis, (10) between group statistical comparisons, (11) point estimates and variability. However, in trials investigating effects of physical exercise interventions true blinding is difficult to achieve for both, participants and for therapists. Therefore, in this review the criteria (5) blinding of subjects and (6) blinding of therapists have not been taken into account (de Morton, 2009) and the highest possible PEDro score that can be achieved is nine instead of 11. If a criterion is clearly met, it is rewarded with one point and is rated as “low risk of bias.” If a criterion is not fulfilled, it is rated as “high risk of bias” and no point is given. If the respective study does not share the information needed for rating, it is rated as “unclear risk of bias.” The initial level of agreement between the ratings by the authors was excellent [intraclass correlation coefficient [ICC] = 0.93]. Discrepancies were solved by discussion and checking the full text.

### Moderator Analysis

#### Exercise Intensity

Potential moderation of exercise intensity was investigated. Intensities of acute exercise were classified into (1) light, (2) moderate, and (3) vigorous exercise intensity. An acute aerobic exercise session was defined as light, if the article reported a rating of perceived exertion between 8 and 10 on the Borg scale, a heart rate between 40 and 55% of maximum heart rate, a heart rate between 20 and 40% of heart rate reserve, or an oxygen uptake between 20 and 40% of maximum oxygen uptake. An acute aerobic exercise session was defined as moderate, if the article reported a rating of perceived exertion between 11 and 13 on the Borg Scale, a heart rate between 56 and 70% of maximum heart rate, a heart rate between 41 and 60% of heart rate reserve, or an oxygen uptake between 41 and 60% of maximum oxygen uptake. An acute aerobic exercise session was defined as vigorous, if the article reported a rating of perceived exertion between 14 and 16 on the Borg Scale, a heart rate between 71 and 90% of maximum heart rate, a heart rate between 61 and 85% of heart rate reserve, or an oxygen uptake between 60 and 85% of maximum oxygen uptake (Norton et al., 2010). An acute resistance exercise session was defined as light, if the article reported an exercise intensity of 61–70% of the participant's individual repetition maximum. It was defined as moderate, if the article reported an exercise intensity of 71–80% of the participants‘ individual repetition maximum. Finally, an acute resistance exercise session was defined as vigorous, if the article reported an exercise intensity of 81–100% of the participants‘ individual repetition maximum (Jovanović and Flanagan, 2014).

#### Type of Exercise

Included studies were subdivided in two different subgroups based on whether an (1) aerobic exercise session or a (2) resistance exercise session was applied.

#### Age

Studies were divided into those that examined (1) young adults (18–34 years of age) and those that examined (2) older adults (35+ years of age).

#### Control Group

The included studies were subdivided according to the used control group paradigm. According to Pontifex et al., we distinguished (1) active control, (2) cognitive engaged control, and (3) passive control groups. Active control groups are physically active, but at such a low intensity, that there is no increase in heart rate. Passive control groups are physically inactive and non-supervised. Cognitive engaging control groups are instructed to perform activities with cognitive stimulation, like watching TV, reading a book, or scrolling on a smartphone.

### Data Analysis

For data analyses, “R” software with the “meta” package (Schwarzer and Carpenter, 2015) was used. As primary meta-analysis, we examined the effect of acute exercise on subsequent set shifting performance in healthy adults. We calculated the “bias corrected Hedges' g standardized mean difference” (SMD) between control group and exercise group at post-intervention for each study. The SMDs were pooled across studies using a random effects model. Interpretation of the size of pooled SMD followed Cohen's classification. Consequently, SMD values of 0.2, 0.5, and 0.8 were interpreted as small, moderate, and large effect sizes, respectively (Cohen, 2013). We quantified “between study heterogeneity,” calculating the T^2^ and the Higgins' *I*^2^ statistic. Higgins' *I*^2^ values of 25, 50, and 75% were interpreted as low, moderate and large proportion of between-study heterogeneity, respectively (Higgins et al., 2003). We conducted sensitivity analyses by excluding studies rated “high risk of bias” on PEDro scale items. Based on subgroup analyses, we examined the moderation potential of the above-described variables.

## Results

### Study Selection

**Figure 1:**
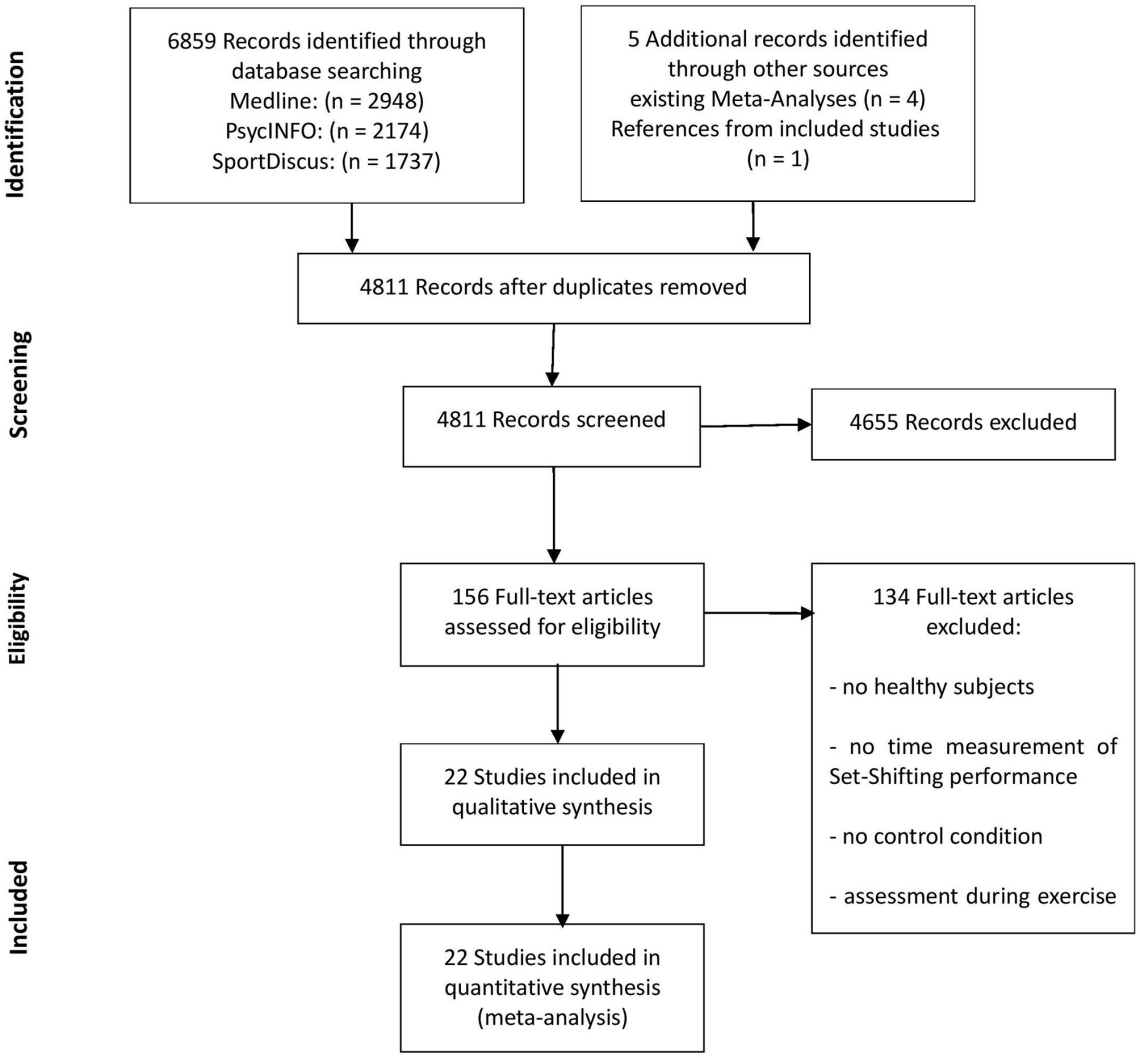
Study selection flow chart according to the PRISMA guidelines.

Through electronic database search, 6,859 publications were identified. The search of the references of recent meta-analyses (Chang et al., 2012; Verburgh et al., 2014; Ludyga et al., 2016) and included studies resulted in 5 additional records. After duplicate removal, titles and abstracts of 4,811 publications were screened. Four thousand, six hundred fifty-five records were excluded because the above defined eligibility criteria for inclusion in this review were not met. One-hundred and fifty-six publications remained, full texts were obtained and read. At this point, 134 studies were excluded for the following reasons: no healthy subjects, no measurement of set shifting performance, no control condition, assessment during exercise. Twenty-two studies, comprising the data from 1,900 participants, were included in a qualitative synthesis and meta-analysis. An overview of the selection process is provided as a PRISMA diagram in Figure 1.

### Characteristics of Studies

**Figure 2:**
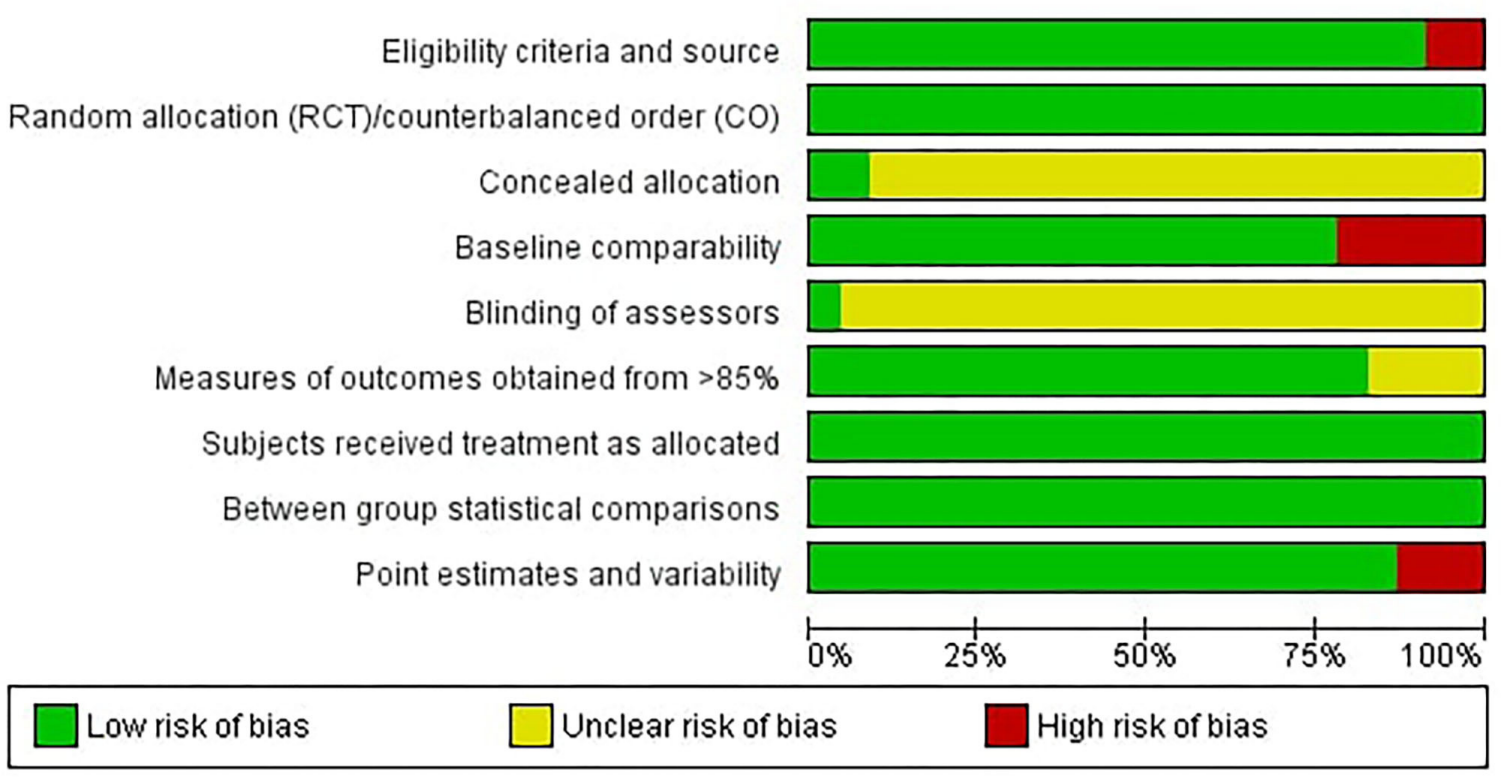
Overview of the PEDro rated study quality (green = low risk of bias, yellow = unclear risk of bias, red = high risk of bias).

Of the 22 included studies, nine studies applied a randomized controlled crossover and 13 studies applied a randomized controlled design. Twelve studies had multiple exercise treatment arms eligible for inclusion in this meta-analysis. Set shifting measures reported in included studies were: averaged or absolute reaction time to complete a defined number of trials, or the number of trials participants processed within a defined time frame. On average, the studies reached a PEDro summary score of 6.52 (SD = 0.93), which relates to a good average methodological quality of included studies (Teasell et al., 2007). An overview of the PEDro rated study quality is depicted in Figure 2. The PEDro ratings for each item and each included study can be found in the Supplementary Material to this article. An extensive summary of characteristics and PEDro summary scores of included studies can be found in Table 1.

**Table 1:** Overview of studies included into meta-analysis on the aftereffect of acute exercise on set shifting performance in healthy adults.

**Study**	**N/Age**	**Study design**	**Exercise treatment**	**Control group treatment**	**Test/Timing of administration**	**Type of variable to measure cognitive flexibility**	**PEDro summary score**
Aguirre-Loaiza et al. (2019)	Gender not reported 21.15 ± 2.21 years	RCT	• Modality: cycling • Description: warm up 60–65% HRmax between 60 and 90 RPM, six sections of 4 min with six peaks of 85% HRmax between 90 and 100 RPM, and active recovery at 75% HRmax, cooldown 60% HRmax between 50 and 70 RPM • Type of exercise: aerobic • Intensity: 75–85% of HRmax ≙ “vigorous” • Duration: 45 min. or 35 min.	• Modality: passive • Description: resting • Duration: 45 min. or 30 min.	TMT Timing of administration not reported	RT	7/9
Alves et al. (2012)	42 (f) 52 ± 7.3 years	Crossover	• Modality: walking • Description: no further information reported • Type of exercise: aerobic • Intensity: 50–60% of HR reserve ≙“moderate” • Duration: 30 min.	• Modality: active • Description: listening to instructions, then low-intensity active stretching exercise • Duration: 30 min.	TMT Timing of administration not reported	RT Accuracy	5/9
Bae and Masaki (2019)	15 (f)/14 (m) 21.4 ± 1.2 years	Crossover	• Modality: cycling • Description: 30 min of self-paced motor-driven treadmill exercise • Type of exercise: aerobic • Intensity: 70% of HRmax ≙ “moderate” • Duration: 30 min.	• Modality: cognitive engagement • Description: reading • Duration: 30 min.	Task switching test Timing of administration not reported	RT (switch cost) Accuracy	7/9
Barenberg et al. (2015) (a)	24 (f)/25 (m) 24.45 ± 2.72 years	Crossover	• Modality: cycling • Description: 2-min warm-up at 25 W, workload was increased by 25 W every 10 s until exhaustion or pace falling below 60 rpm. 4-min recovery phase at 20 W • Type of exercise: aerobic • Intensity: 70 (rpm) ≙ “vigorous” • Duration: 45 min.	• Modality: cognitive engagement • Description: sitting in a chair and listening to music • Duration: 10 min.	Switch trial test Timing of administration not reported	RT (switch cost)	6/9
Barenberg et al. (2015) (b)	27 (f)/18 (m) 23.02 ± 1.71 years	Crossover	• Modality: cycling • Description: 2-min warm-up at 25 W, workload was increased by 25 W every 10 s until exhaustion or pace falling below 60 rpm. 4-min recovery phase at 20 W • Type of exercise: aerobic • Intensity: 70 (rpm) ≙ “vigorous” • Duration: 45 min.	• Modality: cognitive engagement • Description: sitting in a chair and listening to music • Duration: 10 min.	Switch trial test Timing of administration not reported	RT (switch cost)	6/9
Basso et al. (2015)	51 (f)/34 (m) 20.78 ± 0.46 years	RCT	• Modality: cycling • Description: 5 min. warm up, 50 min of vigorous-intensity aerobic exercise, 5 min. cool-down • Type of exercise: aerobic • Intensity: 85% of HRmax ≙ “vigorous” • Duration: 60 min.	• Modality: cognitive engagement • Description: watching TV • Duration: 60 min.	TMT 30, 60 min. after exercise cessation	RT	7/9
Brush et al. (2016)	14 (f)/14 (m) 20.5 ± 2.1 years	Crossover	• Modality: bench press, shoulder press, dumbbell rows, alternating bicep curls, triceps pushdowns, leg extensions, and leg curls • Description: three sets of 10 repetitions, 120 s rest between sets and exercises • Type of exercise: resistance • Intensity: 40%,70% and 100% of 10-RM ≙ “light,” “moderate” and “vigorous” • Duration: 45 min.	• Modality: cognitive engagement • Description: watching video • Duration: 45 min.	Dimension-Switching Task, Plus–Minus Task 15 min. after exercise cessation	RT accuracy	5/9
Chang and Etnier (2009)	27 (f)/14 (m) 49.1 ± 8.73 years	RCT	• Modality: right-arm curl, left-arm curl, dumbbell rowing-right hand, dumbbell rowing-left hand, dumb- bell lateral raise, and bench press • Description: two sets of 10 repetitions for six exercises • Type of exercise: resistance • Intensity: 75% of their theoretical 1-RM ≙ “moderate” • Duration: 45 min.	• Modality: cognitive engagement • Description: reading • Duration: 45 min.	TMT Timing of administration not reported	RT	7/9
Chen et al. (2018)	26 (f)/19 (m) 57.67 ± 5.06y	Crossover	• Modality: cycling • Description: 5 min. warm up, 10 min., 20 min. or 45 min. exercise, 5 min. cooldown • Type of exercise: aerobic • Intensity: 65–70% of HR reserve ≙ “vigorous” • Duration: 20 min., 30 min. or 55 min.	• Modality: cognitive engagement • Description: reading • Duration: 30 min.	Task Switching Test Timing of administration not reported	RT (switch cost) Accuracy	7/9
Coles and Tomporowski (2008)	18 (f+m) 22.2 ± 1.6 years	Crossover	• Modality: cycling • Description: 5-min warm-up, 30-min period, 5-min cool-down period • Type of exercise: aerobic • Intensity: 30% → 60% → 30% of VO_2_max ≙ vigorous • Duration: 40 min.	• Modality: cognitive engagement • Description: sitting • Duration: 40 min.	Visual switch task 5 min. after exercise cessation	RT (switch cost)	5/9
				• Modality: cognitive engagement • Description: watching TV • Duration: 40 min.			
Córdova et al. (2009)	48 (f) 63.44 ± 1.08 years	RCT	• Modality: cycling • Description: 5 min warm-up, 20 min exercise • Type of exercise: aerobic • Intensity: 60%,90% or 110% of the AT ≙ “light,” “moderate” and “vigorous” • Duration: 25 min.	• Modality: passive • Description: sitting • Duration: 25 min.	TMT •8 min. after exercise cessation	RT	7/9
Crush and Loprinzi (2017)	239 (f)/113 (m) 21.05 ± 0.21 years	Crossover	• Modality: running • Description: exercising on a treadmill • Type of exercise: aerobic • Intensity: 40% and 59% of HRreserve≙ “moderate” • Duration: 10 min.	• Modality: passive• Description: no control treatment, baseline testing• Duration: /	TMT 5 min, 15 min., or 30 min. after exercise cessation	RT	6/9
Douris et al. (2018)	24 (f)/14 (m) 23.7 ± 1.8 years	RCT	• Modality: cycling • Description: cycling on a Monarch 818E Lower Body Ergometer • Type of exercise: aerobic • Intensity: 60 to 70% of HRmax ≙ “vigorous” • Duration: 30 min.	• Modality: passive • Description: sitting • Duration: 30 min.	TMT Timing of administration not reported	RT	7/9
Frith et al. (2017)	12 (f)/10(m) 21.9 ± 2.4 years	RCT	• Modality: jogging • Description: jog on treadmill, 5 min at self-selected intensity, 5 min at a self-selected faster pace, 5 min at a self-selected hard pace • Type of exercise: aerobic • Intensity: RPE of 11–12 → RPE of 13–15 → RPE of 16–20 ≙ “vigorous” • Duration: 15 min.	• Modality: passive • Description: sitting • Duration: 20 min.	TMT Timing of administration not reported	RT (switch cost)	7/9
Hwang et al. (2016)	32 (f)/26 (m) 23.59 ± 1.06 years	RCT	• Modality: running • Description: running on a treadmill, 2-min warm up, 5-min increased speed to reach target HR, 10-min running at target HR, 3-min cool down. • Type of exercise: aerobic • Intensity: 85–90% VO2max ≙ “vigorous” • Duration: 20 min.	• Modality: passive • Description: sitting • Duration: 20 min.	TMT 10 min. after exercise cessation	RT	7/9
Jaffery et al. (2018)	64 (f)/24 (m) 21.5 ± 0.5 y	RCT	• Modality: walking • Description: walk at a self-selected brisk walking pace • Type of exercise: aerobic • Intensity: “light” • Duration: 5 min.	• Modality: passive • Description: sitting • Duration: 10 min. or 5 min.	TMT 5 min. after exercise cessation	RT	7/9
Kujach et al. (2020)	36 (m) 21.35 ± 0.5 years	RCT	• Modality: cycling • Description: 5-min warm up at 1.5 Watts × kg−1 of BM. interval exercise, six sets of 30 s of “all out” sprint cycling exercise. Flywheel resistance equaled 0.075 kG × kg−1 of BM (i.e., Wingate test based) which corresponded to 7.5% of each individual's BM, interval rest periods between the 30-s bouts were 4 min and 30 s • type of exercise: aerobic • Intensity: >100% VO_2_max ≙ “vigorous” • Duration: till exhaustion	• Modality: passive • Description: resting • Duration: not reported	TMT 20 min. after exercise cessation	RT	7/9
Murray and Russoniello (2012)	60 (f)/60 (m) 20.86 ± 0.27 years	RCT	• Modality: cycling • Description: pedal a stationary bike • type of exercise: aerobic • Intensity: mean HR intensity of 75.46 % (SD = 8.26) ≙ “vigorous” • Duration: 30 min.	• Modality: cognitive engagement • Description: monitor and motivate other participants • Duration: 30 min.	TMT Timing of administration not reported	RT	6/9
Naderi et al. (2018)	24 (f)/24 (m) 63.58 ± 0.34 years	RCT	• Modality: chest presses, shoulder presses, high pull-downs, rowing, alternating biceps curls, leg extensions, leg curls, and leg presses • Description: 5–10 min. warm-up with light aerobic activity and general stretching exercise, three sets of 10 repetitions. Rest intervals 30 s and 90 s. • Type of exercise: resistance • Intensity: 70% of 10-RM ≙ “light” • Duration: 45 min.	• Modality: cognitive engagement• Description: watching video • Duration: 45 min.	•More-odd task 15 min. after exercise cessation	RT (switch cost) Accuracy	5/9
Oberste et al. (2016)	37 (f)/84 (m) 23.92 ± 0.09 years	RCT	• Modality: cycling • Description: 5 min. warm up while cycling at 25 W, constantly 70 rpm. Then increasing W until participants reached targeted HR range • Type of exercise: aerobic • Intensity: 45–50%, 65–70%, or 85–90% of HRmax. ≙ “light,” “moderate,” and “vigorous” • Duration: 35 min.	• Modality: active • Description: low level self-massage session using a foam roll guided by an experienced instructor • Duration: 35 min.	TMT 10 min. after exercise cessation	RT	9/9
Schwarck et al. (2019)	39 (m) 23.33 ± 3.23 years	RCT	• Modality: cycling • Description: 30 min. treadmill cycling, 5 min. cooldown or 5 min warmup, five 2 min intervals, 5 min. cooldown • Type of exercise: aerobic • Intensity: 40–59% VO2max and 90% VO_2_max ≙ “moderate” and “vigorous” • Duration: 35 min.	• Modality: passive • Description: sitting • Duration: 10 min.	TMT 10 min. after exercise cessation	RT	7/9
Slusher et al. (2018)	13 (m) 23.62 ± 1.06 years	RCT	• Modality: cycling • Description: 10 min. warm-up at 50W on a cycle ergometer. Immediately following: low-volume, supramaximal HIIE. The HIIE protocol: 10 maximal bouts of all out pedaling for 20- s against 5.5% of the subject's body weight, separated by a 10-s active rest period • Type of exercise: aerobic • Intensity: 170% of an individual's VO_2_peak ≙ “vigorous” • Duration: 15 min.	• Modality: active • Description: low level self-massage session using a foam roll guided by an experienced instructor • Duration: 35 min.	WCST Immediately after exercise cessation	RT Accuracy	4/9
Wu et al. (2019)	17 (f)/13 (m) 21.17 ± 1.32 years	Crossover	• Modality: cycling • Description: 5 min. warm up, 20 min exercise, 5 min. cooldown • Type of exercise: aerobic • Intensity: 60–70% of HR reserve ≙ “moderate” • Duration: 30 min.	• Modality: cognitive engagement • Description: reading • Duration: 30 min.	Task switching test Timing of administration not reported	RT (switch cost)	7/9
			• Modality: leg press, leg extension, chest press, chest fly, lateral pulldown, right bicep curl, and left bicep curl • Description: 5 min. warm-up, two sets of 8–12 repetitions for approximately 20 min • Type of exercise: resistance • Intensity: 70% of their 10 RM. ≙ “light,” • Duration: 25 min.				

*f, female; m, male; RCT, randomized controlled trial; HRmax, maximum heart rate; W, Watt performance; VO2peak; peak oxygen uptake; RPM, revolutions per minute, TMT, Trail Making Test; RT, reaction time; RM, repetition maximum; AT, anaerobic threshold; BM, baseline maximum*.

### Results of Primary Meta-Analysis

**Figure 3:**
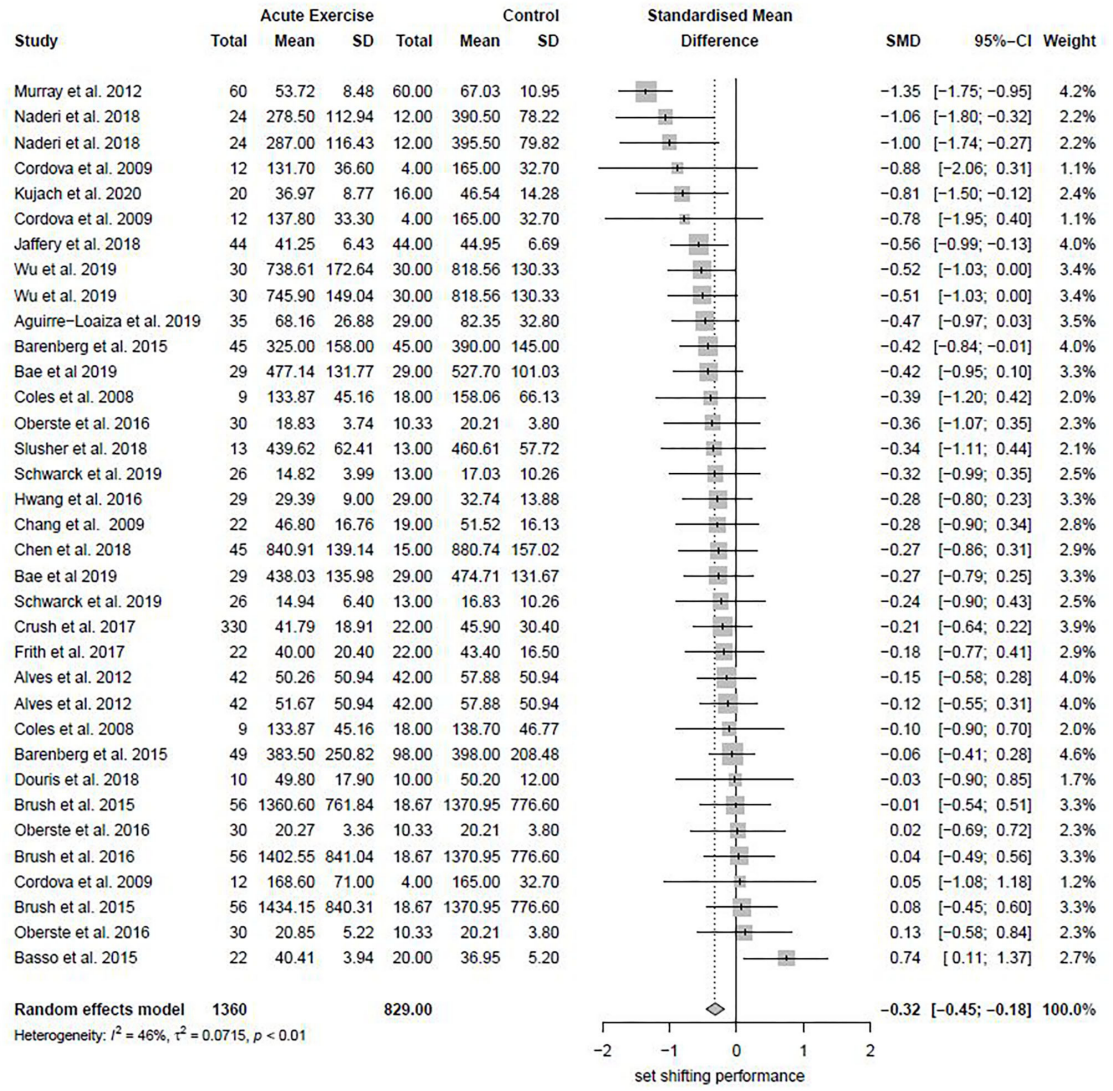
The effect of acute exercise on subsequent set shifting performance compared to control in healthy adults: Overview of all included studies.

It should be noted that negative SMDs represent a beneficial effect of acute exercise compared to control treatment as it reflects a decrease in time needed to complete the task. Thirty-five effect sizes from 22 studies were included in the analysis (1,900 participants). A small to moderate beneficial effect of acute aerobic exercise on subsequent set shifting performance was revealed (k = 35, Hedges' g = −0.32, 95% CI −0.45 to −0.18, *p* < 0.0001). A moderate heterogeneity amongst the included effect sizes was detected (T^2^ = 0.0715, I^2^ = 46.4%). Q-test revealed statistical significance of heterogeneity (*p* < 0.0016), supporting the assumption of random effects. No evident outliers were identified. An overall forest plot is presented in Figure 3.

### Sensitivity Analysis

One or more included studies were rated “high risk of bias” for the PEDro scale items “eligibility criteria and source,” “baseline comparability, and “point estimates and variability.” If the studies that did not give sufficient information concerning eligibility criteria and source were excluded from analysis, the aggregated effect did not decrease while heterogeneity slightly increased compared to initial analysis (k = 32, Hedges' g = −0.39, 95% CI = −0.59 to −0.18, *p* < 0.001, T^2^ = 0.2521, *I*^2^ = 75.5%). If the studies that did not show comparable baseline results were excluded from analysis, the aggregated effect increased substantially. Again, heterogeneity increased (k = 30, Hedges' g = −0.43, 95% CI = −0.68 to −0.23, *p* < 0.001, T^2^ = 0.2931, I^2^ = 77.5%). If the studies that did not provide point estimates and/or variability were removed from the analysis, the aggregated effect turned out to be larger while the heterogeneity increased (k = 30, Hedges' g = −0.46, 95% CI = −0.68 to −0.23, *p* < 0.001, T^2^ = 0.2931, I^2^ = 75.5%). If all studies that were rated “high risk of bias” in at least one item of the PEDro scale were excluded from analysis, the aggregated effect increased just as the heterogeneity did (k = 21, Hedges' g = −0.52, 95% CI = −0.84 to −0.19, *p* < 0.001, T^2^ = 0.4476, I^2^ = 81.1%).

### Results of Moderator Analyses

Table 2 provides a summary of the moderator analyses. The forest plots to subgroup analyses, which are not depicted in this article, are provided in the Supplementary Material to this article.

**Table 2:** Subgroups analyses.

	**k**	**Hedges‘ g**	**95% CI**	**Heterogeneity**	**Test for subgroup difference**
Primary meta–analysis	35	−0.32	−0.45 to −0.18	T^2^ = 0.0715, I^2^ = 46.4%	
**EXERCISE INTENSITY:**					
Light	7	−0.51	−0.83 to −0.19	T^2^ = 0.0742, I^2^ = 37.8%	Q_between_ = 1.83, df = 2, *p =* 0.4014
Moderate	9	−0.24	−0.50 to −0.03	T^2^ = 0.0742, I^2^ = 0%	
Vigorous	19	−0.29	−0.47 to −0.11	T^2^ = 0.0742, I^2^ = 61.7%	
**TYPE OF EXERCISE:**					
Aerobic exercise	27	−0.32	−0.48 to −0.17	T^2^ = 0.0754, I^2^ = 47.6%	
Resistance exercise	8	−0.30	−0.58 to −0.03	T^2^ = 0.0754, I^2^ = 47.4%	Q_between_ = 0.02, df = 1, *p =* 0.8977
**AGE GROUP:**					
Young adults	26	−0.29	−0.44 to −0.13	T^2^ = 0.0764, I^2^ = 23.1%	
Older adults	9	−0.42	−0.72 to −0.13	T^2^ = 0.0764, I^2^ = 52.8%	Q_between_ = 0.60, df = 1, *p =* 0.4383
**TYPE OF CONTROL GROUP:**					
Active control group	6	−0.13	−0.46 to.20	T^2^ = 0.0742, I^2^ = 0%	
Passive control group	13	−0.38	−0.62 to −0.14	T^2^ = 0.0742, I^2^ = 0%	
Cognitive engaging control group	16	−0.34	−0.53 to −0.15	T^2^ = 0.0742, I^2^ = 71.7%	Q_between_ = 1.49, df = 2, *p =* 0.4742

*k, number of effect size estimates, Hedges' g, pooled effect sizes estimate; CI, confidence interval, df, degrees of freedom, p, level of significance*.

#### Subgroups Analysis for “Exercise Intensity”

Six studies chose to examine the effects of a light exercise intensity on subsequent set shifting performance (k = 7, Hedges' g = −0.51, 95% CI −0.83 to −0.19, T^2^ = 0.0742, I^2^ = 37.8%). Eight studies had their participants perform an exercise bout of moderate intensity prior to measurement of set shifting performance (k = 9, Hedges' g = −0.24, 95% CI −0.50 to 0.03, T^2^ = 0.0742, I^2^ = 0%). Nineteen studies investigated the effects of a vigorous exercise intensity on subsequent set shifting performance (k = 19, Hedges' g = −0.29, 95% CI −0.47 to −0.11, T^2^ = 0.0742, I^2^ = 61.7%). No significant difference between subgroups was detected (Q-between = 1.83, df = 2, *p* = 0.4014).

#### Subgroups Analysis for “Type of Exercise”

In 19 included studies, participants performed a bout of acute aerobic exercise (k = 27, Hedges' g = −0.32, 95% CI −0.48 to −0.17, T^2^ = 0.0754, I^2^ = 47.6%) while four studies subjected their participants to an acute bout of resistance exercise (k = 8, Hedges' g = −0.30, 95% CI −0.58 to −0.03, T^2^ = 0.0754, I^2^ = 47.4%). Subgroups summary effects did not differ significantly from each other (Q-between = 0.02, df = 1, *p* = 0.8977).

#### Subgroups Analysis for “Age”

**Figure 4:**
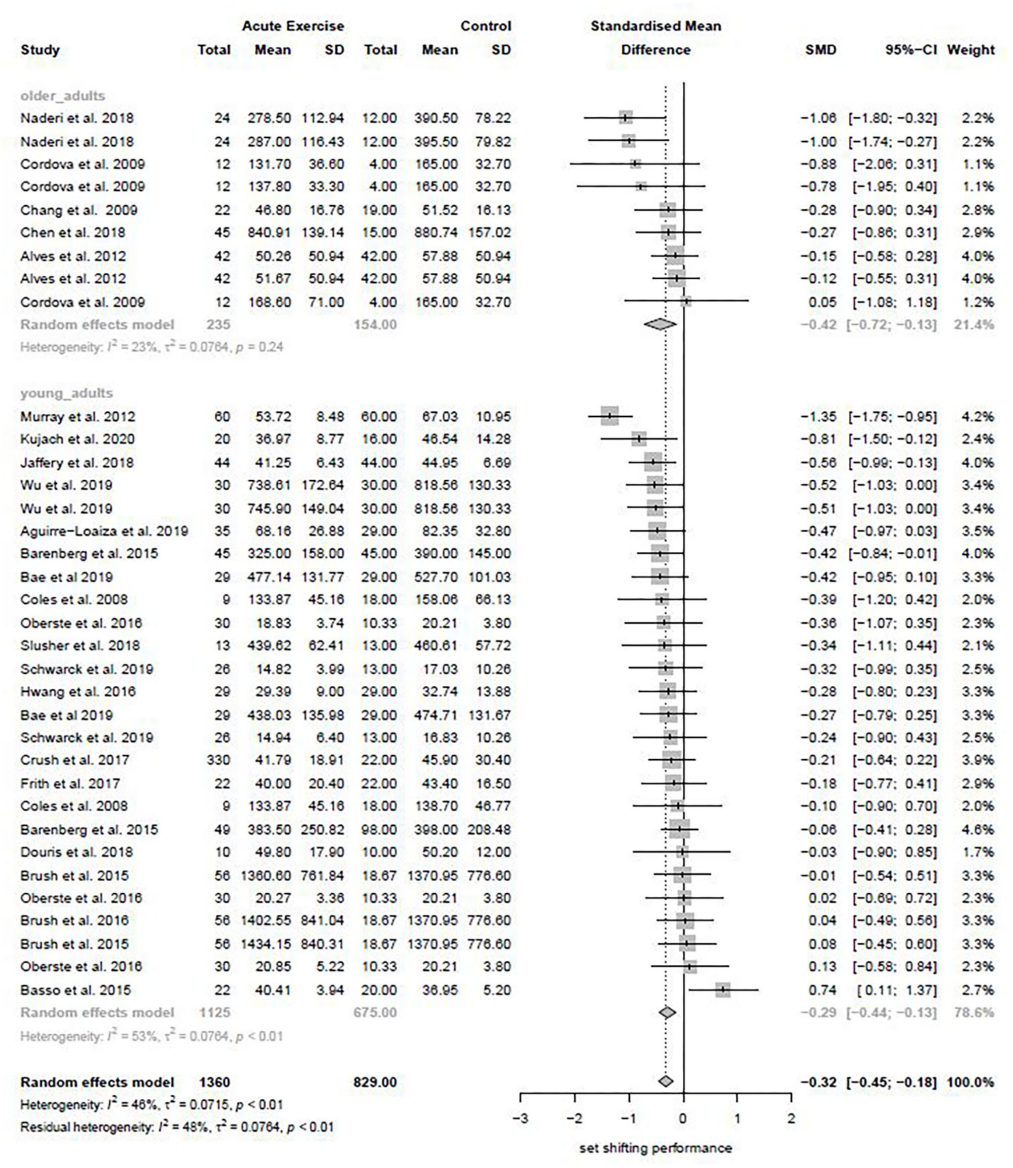
Subgroup Analysis for age group.

Sixteen studies included in this meta-analysis investigated the effect of acute exercise on subsequent set shifting performance in younger adults (age 18–34) (k = 26, Hedges' g = −0.29, 95% CI −0.44 to −0.13, T^2^ = 0.0764, I^2^ = 23.1%). In contrast, five studies examined the effect of acute exercise on subsequent set shifting performance in older adults (age 35+) (k = 9, Hedges' g = −0.42, 95% CI −0.72 to −0.13, T^2^ = 0.0764, I^2^ = 52.8%). The forest plot to this subgroup analysis is depicted in Figure 4. The difference between the subgroups' summary effects did not reach statistical significance (Q-between =0.60, df = 1, *p* = 0.4383).

#### Subgroups Analysis for “Control Group”

Three studies included in this meta-analysis decided to use a physically active control group as a standard of comparison to the acute exercise condition (k = 6, Hedges' g = −0.13, 95% CI −0.46 to 0.20, T^2^ = 0.0742, I^2^ = 0%). Twelve studies used a cognitive engaging control treatment (k=16, Hedges' g = −0.34, 95% CI −0.53 to −0.15, T^2^ = 0.0742, *I*^2^ = 71.7%). Nine studies compared the acute exercise condition with a physically inactive and therefore passive control treatment (k=13, Hedges' g = −0.38, 95% CI −0.62 to −0.14, T^2^ =0.0742, *I*^2^ = 0%). The difference between subgroups summary effects was not statistically significant (Q-between = 1.49, df = 2, *p* = 0.4742).

## Discussion

This meta-analysis showed significant small to moderate effects of acute aerobic exercise on subsequent set shifting performance in healthy adults (g = −0.32). Sensitivity analyses show that this effect is unlikely due to bias of individual studies. Whether the small to moderate benefit in time when shifting between two different tasks is of practical relevance is difficult to evaluate. In special situations, where fast set-shifting performance is required, as in professional e-sports, a small speed advantage might make a decisive difference. By contrast, in the context of average everyday work life in an office for example, these exercise-induced benefits will hardly mean a noticeable improvement.

In addition, this meta-analysis investigated the influence of potential moderators. Light exercise intensity showed a moderate effect on subsequent set shifting performance (g = −0.51). However, moderate (g = −0.24) and vigorous exercise intensity (g = −0.29) showed only a small to moderate effect. This result may be explained by the different physiological responses to acute exercise at light and moderate/vigorous intensity. Moderate and vigorous exercise induce more fatigue and dehydration, which are assumed to have a disadvantageous effect on subsequent cognitive performances (Cian et al., 2001). Results of existing meta-analyses investigating the effect of the exercise intensity on executive function are inconsistent. Contrasting to the present analysis, McMorris and Hale (2012) showed a bigger improvement in inhibition, working memory and set shifting performances following an exercise session of moderate intensity than of light or vigorous intensity (McMorris and Hale, 2012). In contrast, Loprinzi and Kane (2015) could not show any differences between a light, moderate or vigorous exercise intensity on set shifting performance at all (Loprinzi and Kane, 2015). While it still remains unclear which exercise intensity should be chosen to achieve the highest improvement in cognitive performances in general, this meta-analysis suggests that a light exercise intensity leads to the highest increase in subsequent set shifting performance.

The comparison of a single bout of aerobic exercise with a single bout of resistance exercise resulted in nearly identical beneficial effects on subsequent set shifting performance. Acute aerobic (g = −0.32) and resistance exercise (g = −0.30) both have a small to moderate beneficial effect on subsequent set shifting performance. This indicates that the previously mentioned differences regarding physical demands do not seem to play a decisive role in the extent to which each type of exercise has an influence on set shifting performance. Therefore, the focus seems to lie on the subsequent physical effects that both types of exercise have in common. Both lead to an increase of cerebral blood flow, which is considered to facilitate waste clearing and corresponding metabolic resource availability. Previous studies support the idea that these effects may ease cognitive processing (Delp et al., 2001; Pereira et al., 2007). Furthermore, both types of exercise lead to a release of catecholamines, which is seen to have an influence on set shifting (Robbins and Roberts, 2007; Bondi et al., 2010), and working memory (McMorris et al., 2016) as well. There is however almost no difference in the extent of the effects both types of exercise have on subsequent set shifting performance.

Although not statistically significant, the results revealed that older adults (g = −0.42) benefit noticeably more from an acute exercise bout than younger adults (g = −0.29) do. One potential explanation for these differences could be different baseline performances in young healthy and older adults. Previous studies showed the most pronounced benefits of acute exercise on executive function, especially on inhibition and working memory, in individuals with low baseline cognitive performance (Sibley and Beilock, 2007; Drollette et al., 2014). According to statements by Zelazo et al. (2004) there is a “rise and fall” of executive function across the human life span, which indicates peak performance during young adulthood and lower performance in older adulthood (Zelazo et al., 2004). Therefore, older adults, with lower baseline performance, might benefit more from acute exercise than young adults with higher baseline set shifting performance. A possible reason for the reduced increase in set shifting performance following a single bout of acute exercise in younger adults is the occurrence of a ceiling effect. Since cognitive performance is expected reaching its peak in the age group of young adults (Zelazo et al., 2004), there seems less room for improvement (Kamijo et al., 2007; O'Leary et al., 2011; Bullock and Giesbrecht, 2014). The results of this meta-analysis with respect to the participants' age were in accordance with these assertions regarding cognitive performance in general. Therefore, it can be assumed that they hold true for the specific subdomain set shifting as well.

Acute exercise led to a moderately higher set shifting performance when compared to passive (g = −0.38) or cognitive engaged (g = −0.34) control groups. By contrast, there was only a very small improvement when acute exercise was compared to active control groups (g = −0.13). These differences in subgroups‘ effect sizes could be explained by the potential occurrence of a placebo effect. In studies investigating the effect of acute physical activity, participants are aware of the physical demands they are being exposed to. Therefore, participants of the active control group are more likely to believe that they are part of the experimental condition, which controls better for expectations of cognitive benefits between the experimental and control group. Conversely, it is more likely for the participants in the passive control group to realize that they are part of the control condition. Therefore, differences in expectations for cognitive benefits between experimental and passive/cognitive engaged control groups are perceivable. Expectations play a key role in the placebo effect (Brown, 2015). The results of this analysis indicate that the magnitude of the effect of acute exercise on set shifting might be overestimated due to Placebo-effect. However, it should also be noted that the use of a placebo or an active control group in a trial might also lead to reduced effects in treatment groups. Negative expectations of the participants due to the chance of receiving the placebo are responsible for that. This effect has been named the “lessebo effect” (Mestre et al., 2014). Despite these differences of effect sizes in each subgroup, none of these results could be regarded as statistically significant. This might be explained by the small number of included effect sizes (35 effect sizes from 22 studies), and the resulting low statistical power. However, it diminishes the generalizability of the here achieved results.

The here presented results must be interpreted against the background of limitations. The results of this meta-analysis are only valid within the boundaries of the stated in- and exclusion criteria. Moreover, this meta-analysis was not able to differentiate between local and global switch cost as a separately primary endpoint. Since only three (Coles and Tomporowski, 2008; Barenberg et al., 2015; Naderi et al., 2018) of the 22 included studies used a variation of the Task-Switching Paradigm as neuropsychological test and therefore could report results for either global or local switch cost, a quantitative analysis for each variable was not possible. Future studies in this field should apply neuropsychological tests that allow a differentiation between local and global switch cost to enable further knowledge in this scientific field.

Another limitation is that meta-analytic subgroups analysis is observational. This means that randomization of participants in single studies does not apply to distinguishing between subgroups of studies. Therefore, systematic differences between subgroups concerning other factors than the subgroup defining cannot be ruled out. Several included studies conducted the neuropsychological testing of set shifting performance repeatedly for different time intervals after exercise cessation. To avoid severe issues with double counts and unit-of-analysis-error in our analysis, these multiple treatment arms were combined to a single pair-wise comparison following Higgins and Deeks' recommendations (Higgins and Deeks, 2011). Therefore, this meta-analysis could not investigate the influence of the different time intervals of test administration on set shifting performance. Future studies should continue to systematically investigate potential moderators with regards to the timing of test administration. This meta-analysis does not give a final answer on the internal validity of research on acute exercise induced benefits for set shifting performance in healthy adults. Potential moderation of widespread methodological shortcomings in that field, like e.g., “concealed allocation” and “blinding of assessors” remain unclear. Future meta-analyses should investigate the moderating influence of these factors. They should also apply a higher methodological standard to increase their internal validity. Also, a two, or more, factor analysis that investigates potential interaction effects between moderators is beyond the scope of our article. Also, lacking theory-driven approaches to the effects of exercise on cognition limit advances in this research field. Although research has started to investigate promising neurobiological factors, the underlying neurobiological mechanisms are still unknown. A deeper understanding of these mechanisms is important for more precise hypotheses, which aspects of cognition may be influenced or even immune to physical activity. As the understanding of biological processes responsible for changes in executive functions is still limited, further research on these underlying mechanisms is encouraged. Finally, this meta-analysis only focused on the effect of acute exercise on subsequent set shifting performance. Long-term exercise interventions are likely to lead to more pronounced positive effects because they are comprised of repetitive acute sessions. Future meta-analyses should focus on acute and long-term exercise and investigate the underlying neurobiological mechanisms.

## Conclusion

This meta-analysis shows that a single bout of acute exercise improves subsequent set shifting performance in healthy adults. However, effects are of small size making relevance for everyday life is questionable. Older adults show moderate beneficial effects, but also young adults being at their peak of cognitive performance still benefit from an acute exercise session. The type of exercise does not influence magnitude of effect sizes, which implies that the improvement in subsequent set shifting performance is due to physical adaptations to the exercise, regardless of its type. Moderate and vigorous exercise intensities both lead too small to moderate beneficial effects in subsequent set shifting performance, while a light exercise intensity results in a moderate beneficial effect. Participants who are part of an active control group show higher improvements in set shifting performance than participants of a passive or cognitive engaged control group, which could be a result of an expectation driven placebo effect.

## Author Contributions

MO, SS, WB, and PZ planned the project. MO and SS conducted literature search, data extraction, and risk of bias assessment. Inconsistencies were resolved in consultation with WB and PZ. MO conducted data analyses. MO and SS wrote the manuscript, and concerning underlying neurobiological effects of acute aerobic exercise. SS communicated with study authors in case of missing data. WB supervised. All authors proofread the manuscript.

## Conflict of Interest

The authors declare that the research was conducted in the absence of any commercial or financial relationships that could be construed as a potential conflict of interest.
