# Supplementary material for: Acute Exercise-Induced Set Shifting Benefits in Healthy Adults and Its Moderators: A Systematic Review and Meta-Analysis
Source: Front Psychol. 2021 Jan 29;12:528352. doi: 10.3389/fpsyg.2021.528352 (PMC7879782; doi:10.3389/fpsyg.2021.528352)
Supplement: Supplementary file 1 [file Table_1.DOCX]

Supplementary Material

# PRISMA Checklist

| **Section/topic** | **#** | **Checklist item** | **Reported on page #** |
| --- | --- | --- | --- |
| **TITLE** | | |  |
| Title | 1 | Identify the report as a systematic review, meta-analysis, or both. | p. 1 |
| **ABSTRACT** | | |  |
| Structured summary | 2 | Provide a structured summary including, as applicable: background; objectives; data sources; study eligibility criteria, participants, and interventions; study appraisal and synthesis methods; results; limitations; conclusions and implications of key findings; systematic review registration number. | pp. 2-3 |
| **INTRODUCTION** | | |  |
| Rationale | 3 | Describe the rationale for the review in the context of what is already known. | pp. 3-4 |
| Objectives | 4 | Provide an explicit statement of questions being addressed with reference to participants, interventions, comparisons, outcomes, and study design (PICOS). | pp. 3-4 |
| **METHODS** | | |  |
| Protocol and registration | 5 | Indicate if a review protocol exists, if and where it can be accessed (e.g., Web address), and, if available, provide registration information including registration number. | p. 5 |
| Eligibility criteria | 6 | Specify study characteristics (e.g., PICOS, length of follow-up) and report characteristics (e.g., years considered, language, publication status) used as criteria for eligibility, giving rationale. | pp. 5-6 |
| Information sources | 7 | Describe all information sources (e.g., databases with dates of coverage, contact with study authors to identify additional studies) in the search and date last searched. | p. 6 |
| Search | 8 | Present full electronic search strategy for at least one database, including any limits used, such that it could be repeated. | p. 6 |
| Study selection | 9 | State the process for selecting studies (i.e., screening, eligibility, included in systematic review, and, if applicable, included in the meta-analysis). | pp. 5-6 |
| Data collection process | 10 | Describe method of data extraction from reports (e.g., piloted forms, independently, in duplicate) and any processes for obtaining and confirming data from investigators. | p. 6 |
| Data items | 11 | List and define all variables for which data were sought (e.g., PICOS, funding sources) and any assumptions and simplifications made. | p. 6 |
| Risk of bias in individual studies | 12 | Describe methods used for assessing risk of bias of individual studies (including specification of whether this was done at the study or outcome level), and how this information is to be used in any data synthesis. | pp. 7 |
| Summary measures | 13 | State the principal summary measures (e.g., risk ratio, difference in means). | pp. 8-9 |
| Synthesis of results | 14 | Describe the methods of handling data and combining results of studies, if done, including measures of consistency (e.g., I^2^) for each meta-analysis. | p. 8 |

Page 1 of 2

| **Section/topic** | **#** | **Checklist item** | **Reported on page #** |
| --- | --- | --- | --- |
| Risk of bias across studies | 15 | Specify any assessment of risk of bias that may affect the cumulative evidence (e.g., publication bias, selective reporting within studies). | p. 7 |
| Additional analyses | 16 | Describe methods of additional analyses (e.g., sensitivity or subgroup analyses, meta-regression), if done, indicating which were pre-specified. | p. 8 |
| **RESULTS** | | |  |
| Study selection | 17 | Give numbers of studies screened, assessed for eligibility, and included in the review, with reasons for exclusions at each stage, ideally with a flow diagram. | pp. 8-9, Figure 1 |
| Study characteristics | 18 | For each study, present characteristics for which data were extracted (e.g., study size, PICOS, follow-up period) and provide the citations. | p. 9 and Table 1 |
| Risk of bias within studies | 19 | Present data on risk of bias of each study and, if available, any outcome level assessment (see item 12). | Figure 2, Supplementary material |
| Results of individual studies | 20 | For all outcomes considered (benefits or harms), present, for each study: (a) simple summary data for each intervention group (b) effect estimates and confidence intervals, ideally with a forest plot. | Table 1, Figure 3, Supplementary material |
| Synthesis of results | 21 | Present results of each meta-analysis done, including confidence intervals and measures of consistency. | pp. 10-11, Figures 3,  Supplementary Material |
| Risk of bias across studies | 22 | Present results of any assessment of risk of bias across studies (see Item 15). | Figure 2  Supplementary material |
| Additional analysis | 23 | Give results of additional analyses, if done (e.g., sensitivity or subgroup analyses, meta-regression [see Item 16]). | pp. 10-11 |
| **DISCUSSION** | | |  |
| Summary of evidence | 24 | Summarize the main findings including the strength of evidence for each main outcome; consider their relevance to key groups (e.g., healthcare providers, users, and policy makers). | pp. 11-14 |
| Limitations | 25 | Discuss limitations at study and outcome level (e.g., risk of bias), and at review-level (e.g., incomplete retrieval of identified research, reporting bias). | p. 14 |
| Conclusions | 26 | Provide a general interpretation of the results in the context of other evidence, and implications for future research. | p. 15 |
| **FUNDING** | | |  |
| Funding | 27 | Describe sources of funding for the systematic review and other support (e.g., supply of data); role of funders for the systematic review. | pp. 15-16 |

*From:*  Moher D, Liberati A, Tetzlaff J, Altman DG, The PRISMA Group (2009). Preferred Reporting Items for Systematic Reviews and Meta-Analyses: The PRISMA Statement. PLoS Med 6(7): e1000097. doi:10.1371/journal.pmed1000097

For more information, visit: **www.prisma-statement.org**.

Page 2 of 2

# Search algorithm

Medline, PsycInfo and SportDiscus was searched using the following terms:

(exercise* [Title] OR sport* [Title] OR "physical activity" [Title] OR "physical exertion" [Title] OR "physical training" [Title] OR running [Title] OR jogging [Title] OR walking [Title] OR bicycling [Title] OR "strength training" [Title]) AND (cogniti* [Title] OR "executive function*" [Title] OR “set shifting” [Title] OR "reaction time" [Title] OR attention [Title])

# Supplementary Figures


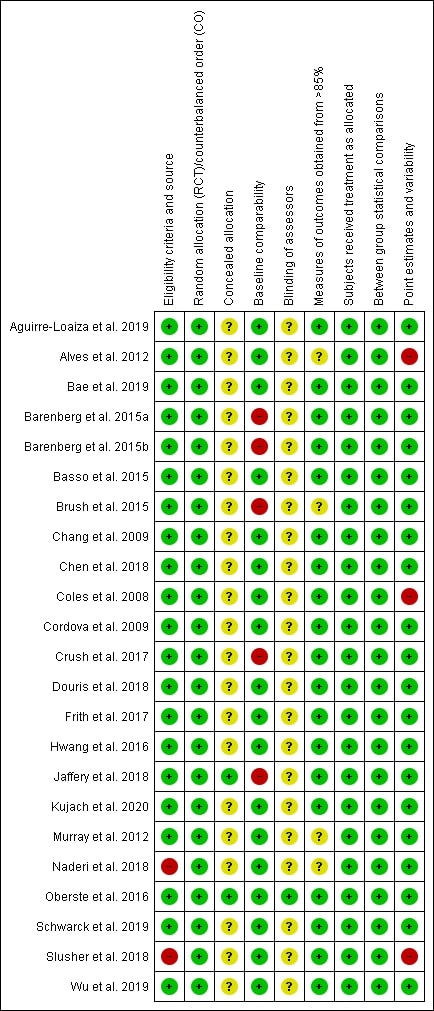


Figure 1. PEDro ratings for each item and each included study


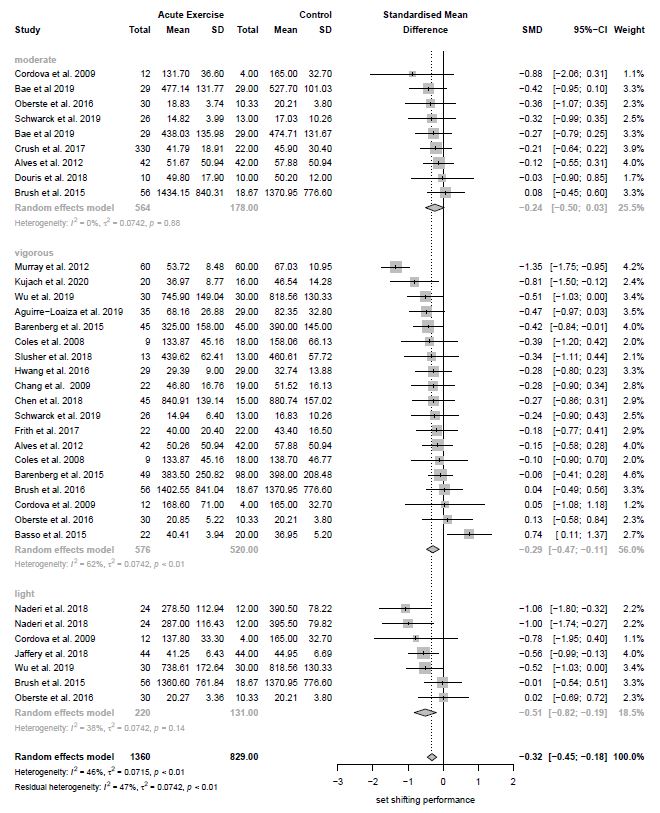


Figure 2. Subgroup analysis for exercise intensity


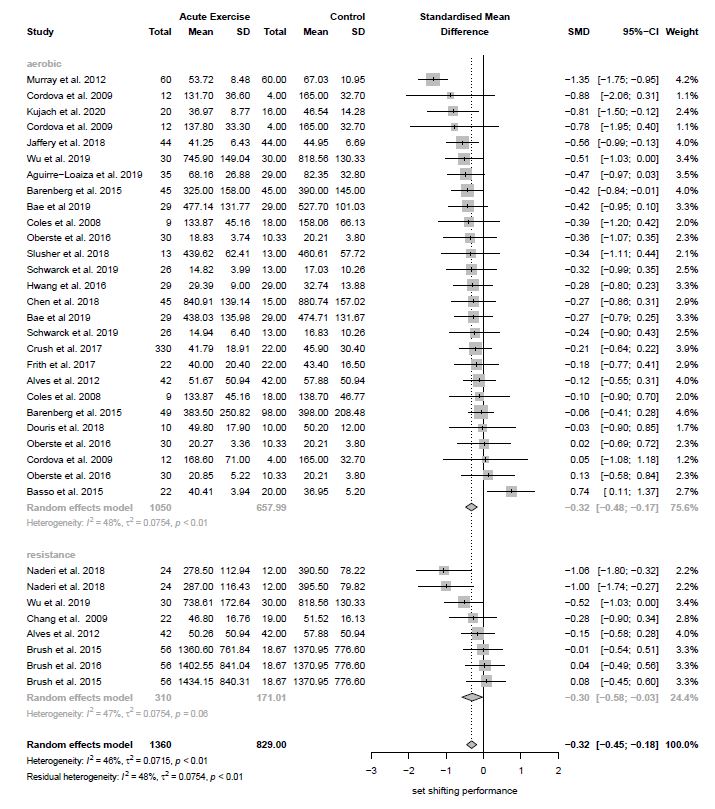


Figure 3. Subgroup analysis for type of exercise


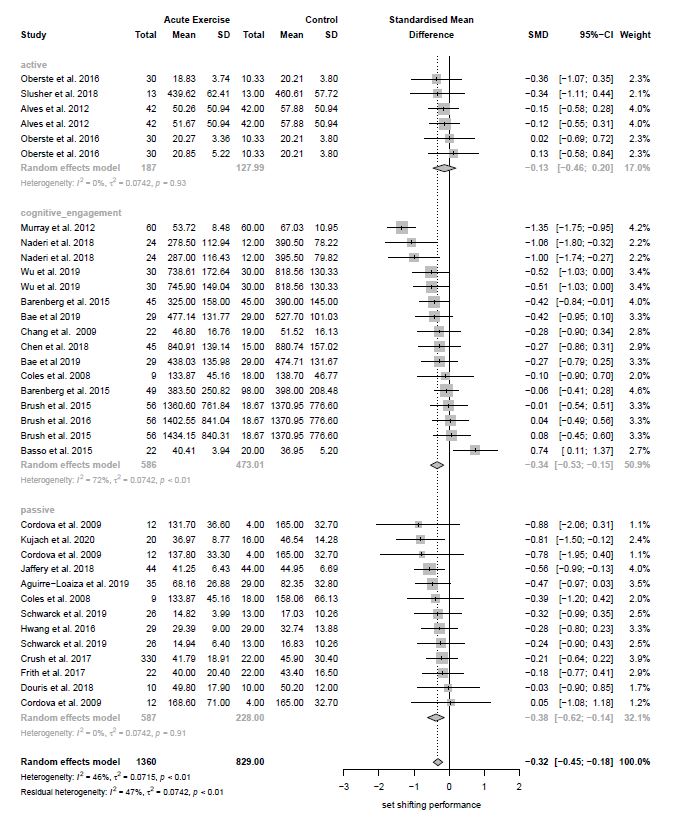


Figure 4. Subgroup analysis for type of control group

Trail Making Test

This test includes two conditions, an A and a B condition. In part A, participants are required to draw a line and connect continuously numbered dots from 1 to 25 in a random pattern on a piece of paper. This condition reflects motor and visual control. In part B, participants are instructed to connect numbers and letters in a given sequence alternately (1—A—2—B—3—C…25—Y). This condition reflects the additional set shifting performance needed to switch between number and letter sequences. Participants fail the test with 7 or more errors in either the A or B condition. Performance in both tests is assessed based on the time needed to complete each condition.

e.g. (Alves et al. 2012)

Wisconsin Card Sorting Task

This test consists of four stimulus cards which can be divided into three categories: the colour of the objects (red, yellow, green, or blue), the number of objects (one, two, three, or four) and the shape of the objects (triangle, star, cross, or circle). The Stimulus cards are presented in a defined order, and the participants are told to match a response card to a corresponding stimulus card based on one of the three mentioned categories. How the cards should be matched is based on a pattern which is unknown to the participants. The only feedback they receive is an audible voice telling them whether the match they made was “right” or “wrong”. The same rule pattern continues until ten cards are matched correctly. Then, the rule pattern changes, and the participants are required to adapt to the new pattern and shift to a new mental set, so they are able to match the cards according to the new rule pattern. The test is completed after a defined number of completed cycles. Performance in this test is assessed based on the total time needed to complete the test.

e.g. (Slusher et al. 2018)

Switch Trial test

This test consists of two single task blocks, which require the participants to classify presented geometric stimuli either by their colour (blue / yellow) or shape (circle / triangle). The stimuli appear one by one and are randomly selected. Participants are told to classify the stimulus as quickly and correctly as possible to make the next stimulus appear. In the following mixed task blocks, participants are required to switch between classifying the colour and the shape of stimuli on every second trial. Performance in this test is assessed based on response latencies of correct answers.

e.g. (Barenberg, Berse, and Dutke 2015)

Dimension Switching Task

The words “left” and “right” are enclosed in either a left or right arrow on a computer screen. An arrow, including either the word “left” or “right”, is presented in the upper or lower half of the screen pointing in either a left or right direction. Depending on where the stimulus is located (upper or lower half of the screen), participants are instructed to perform either the word task or the arrow task. For the word task, participants press a button in the direction indicated by the word which is written on the arrow. For the arrow task, participants press a button in the direction indicated by the arrow’s direction. There are two kinds of blocks. On simple blocks, the arrows are presented always on the same location, requiring the participant to only perform one of the tasks at a time. On mixed blocks, arrows appear in the upper or lower half of the screen, requiring the participant to shift from one set to another set. Performance is assessed based on the reaction time difference between trials from the simple blocks and from the mixed blocks.

e.g. (Boucard et al. 2012)

Plus-Minus Task

This task consists of three lists of 30 numbers in a range from 10 to 99. On the first list, the participants are instructed to add 3 to each number and to write down the answers on a piece of paper. On the second list, they are instructed to subtract 3 from each number. In the third list, they are instructed to alternate between adding 3 and subtracting 3 from the numbers, requiring the participant to shift from one set to another. Participants are told to complete each list as fast and accurately as possible. Performance is assessed by subtracting the mean total time participants needed to complete the first and the second list from the total time on the alternating third list.

e.g. (Boucard et al. 2012)

More Odd Task

This task comprises of a series of numbers from either 1 to 4 or 6 to 9 presented on a computer screen. Block A consists of a defined number of simple task trials. The numbers are colored in black and the participants are told to press the corresponding key to indicate whether the appearing number is greater than or less than 5. Block B consists of a defined number of simple task trials as well, but now the numbers are colored in green. The participants are told to press the corresponding key to indicate whether the appearing number is odd or even. Block C comprises of twice as many mixed task trials and includes both A and B simple task trials. They switch from one to the other on every second trial. The participants are instructed to press the corresponding key to identify whether the number is greater or less than 5 when it appears in black and whether the number is odd or even when it appears in green, requiring the participant to shift from one set to another. Performance is assessed based on the response time difference between the simple task trials and mixed task trials blocks.

e.g. (A. G. Chen et al. 2014)

Visual Switch Task

In this task, the participant is told to respond to a series of a defined number of letter – number pairs (e.g. D – 6) that appear either in the upper or lower half of a computer screen. The location of each pair is predictable for the participants. If the pair is presented in the upper half of the screen, the number of the pair is the relevant category and the participant is needed to determine whether the number is even or odd by pressing the appropriate button. If the pair is presented in the bottom of the screen, the letter of the pair is the relevant category and the participant is needed to determine whether the letter is a consonant or a vowel by pressing the appropriate button. The next pair appears immediately after a response. Performance in this test is assessed based on the total time needed to complete the test.

e.g. (Coles and Tomporowski 2008)

Task Switching Task

In this test, numbers from 1 to 9, excluding 5, are presented on a white screen colored in black with either solid or dotted lines surrounding the digit. The test consists of six blocks comprising two simple task blocks followed by four mixed task blocks. In the first simple task block, participants are instructed to indicate whether the number that appears within solid lines is greater or less than 5. In the second simple task block, the participants are told to indicate whether the number that appears within dotted lines is odd or even. Participants are needed to respond to each stimulus by pressing the corresponding key as fast and correctly as possible. The third to sixth blocks are four identical mixed task blocks. Stimuli in this condition are the same as presented in the first two single task blocks (same digits with solid or dotted lines). Participants are instructed to indicate the magnitude when the number is surrounded with solid lines or to identify the number as odd or even when it is surrounded with dotted lines, requiring the participant to shift from one set to another. Performance is assessed based on the response time difference between the simple task trials and mixed task trials blocks.

e.g. (F.-T. Chen et al. 2018)

**References**

Alves, Christiano Robles Rodrigues, Bruno Gualano, Pollyana Pereira Takao, Paula Avakian, Rafael Mistura Fernandes, Diego Morine, and Monica Yuri Takito. 2012. “Effects of Acute Physical Exercise on Executive Functions: A Comparison between Aerobic and Strength Exercise.” *Journal of Sport and Exercise Psychology* 34 (4): 539–49. https://doi.org/10.1123/jsep.34.4.539.

Barenberg, Jonathan, Timo Berse, and Stephan Dutke. 2015. “Ergometer Cycling Enhances Executive Control in Task Switching.” *Journal of Cognitive Psychology* 27 (6): 692–703. https://doi.org/10.1080/20445911.2015.1024256.

Boucard, Geoffroy K., Cédric T. Albinet, Aurélia Bugaiska, Cédric A. Bouquet, David Clarys, and Michel Audiffren. 2012. “Impact of Physical Activity on Executive Functions in Aging: A Selective Effect on Inhibition among Old Adults.” *Journal of Sport and Exercise Psychology*. https://doi.org/10.1123/jsep.34.6.808.

Chen, Ai Guo, Jun Yan, Heng Chan Yin, Chien Yu Pan, and Yu Kai Chang. 2014. “Effects of Acute Aerobic Exercise on Multiple Aspects of Executive Function in Preadolescent Children.” *Psychology of Sport and Exercise*. https://doi.org/10.1016/j.psychsport.2014.06.004.

Chen, Feng-Tzu, Jennifer L Etnier, Chih-Han Wu, Yu-Min Cho, Tsung-Min Hung, and Yu-Kai Chang. 2018. “Dose-Response Relationship between Exercise Duration and Executive Function in Older Adults.” *Journal of Clinical Medicine* 7 (9). https://doi.org/10.3390/jcm7090279.

Coles, Kathryn, and Philip D Tomporowski. 2008. “Effects of Acute Exercise on Executive Processing, Short-Term and Long-Term Memory.” *Journal of Sports Sciences* 26 (3): 333–44. https://doi.org/10.1080/02640410701591417.

Slusher, Aaron L, Virginia T Patterson, Charles S Schwartz, and Edmund O Acevedo. 2018. “Impact of High Intensity Interval Exercise on Executive Function and Brain Derived Neurotrophic Factor in Healthy College Aged Males.” *Physiology & Behavior* 191 (July): 116–22. https://doi.org/10.1016/j.physbeh.2018.04.018.
